# Impact of low-load blood flow restriction training on knee osteoarthritis pain and muscle strength: a systematic review and meta-analysis of randomized controlled trials

**DOI:** 10.3389/fphys.2025.1524480

**Published:** 2025-03-17

**Authors:** Qiuxiang Lin, Debiao Yu, Yuping Zhang, Xiaoting Chen, Jiawei Qin, Fuchun Wu

**Affiliations:** ^1^ Department of Rehabilitation Medicine, Quanzhou First Hospital Affiliated to Fujian Medical University, Quanzhou, China; ^2^ College of Rehabilitation Medicine, Fujian University of Traditional Chinese Medicine, Fuzhou, China; ^3^ Provincial Clinical Medicine College of Fujian Medical University, Fuzhou, China; ^4^ Department of Rehabilitation Medicine, Fujian Provincial Hospital, Fuzhou, China; ^5^ Department of Rehabilitation Medicine, Fuzhou University Affiliated Provincial Hospital, Fuzhou, China; ^6^ Department of Orthopedics, Quanzhou First Hospital Affiliated to Fujian Medical University, Quanzhou, China

**Keywords:** blood flow restriction training, pain, rehabilitation, knee osteoarthritis, physical function

## Abstract

**Objectives:**

The effectiveness of low-load blood flow restriction training (LL-BFRT) in alleviating symptoms in patients with knee osteoarthritis (KOA) remains inconclusive. This systematic review and meta-analysis aim to comprehensively assess the effects of LL-BFRT compared to conventional resistance training on pain, muscle strength, and functional capacity in individuals with KOA.

**Data sources:**

PubMed, Embase, Web of Science, EBSCO, Scopus, and Cochrane trails were searched.

**Study selection:**

We included randomized controlled trials involving patients with KOA, in which the intervention group underwent LL-BFRT.

**Data extraction:**

Literature quality and risk of bias were assessed using the Physiotherapy Evidence Database (PEDro) scale and the Cochrane Risk-of-Bias Tool (ROB 2). Data were extracted using a predefined table, including outcomes such as pain, quadriceps muscle strength, 30-s sit-to-stand test (30STS) and Timed Up and Go test (TUG).

**Result:**

Ten studies were included in the meta-analysis. The pooled results indicated that, compared to conventional resistance training, LL-BFRT significantly improved knee joint pain [SMD = 0.25, 95%CI (0.02, 0.48), P = 0.03], increased quadriceps muscle strength [SMD = 0.46, 95%CI (0.04, 0.88), P = 0.03], and enhanced performance on the 30s sit-to-stand test (30STS) [WMD = 1.71, 95%CI (0.30, 3.11), P = 0.02]. However, no significant difference was observed in the improvement of the Timed Up and Go test (TUG) [WMD = −0.13, 95%CI (−0.51, 0.24), P = 0.49]. Subgroup analysis revealed that interventions with an occlusion pressure >100 mmHg and a duration ≤6 weeks had a significant impact on pain relief, quadriceps muscle strength, and the 30STS performance. For patients with KOA aged >65 years, LL-BFRT was more effective in alleviating pain, while for patients aged ≤65 years, it demonstrated more significant improvements in quadriceps strength and 30STS performance.

**Conclusion:**

Limited evidence suggests that LL-BFRT may be more effective than conventional resistance training in improving pain, quadriceps muscle strength, and 30STS performance in patients with KOA, while exhibiting a comparable effect on TUG test.

**Systematic Review Registration:**

https://www.crd.york.ac.uk/prospero/#myprospero, identifier CRD42024603542

## Introduction

Pain is a primary symptom of knee osteoarthritis (KOA) and a major reason why patients seek treatment (Jackson et al., 2023). The prevalence of KOA increases with age, with a global estimated prevalence of 3.8% (Cross et al., 2014; Ji et al., 2023). The pain associated with KOA can result in functional limitations and a loss of independence (Giorgino et al., 2023; Hawker, 2019). As KOA progresses, joint replacement surgery may become necessary, further exacerbating the societal and healthcare burden (Riddle and Jiranek, 2015; Costa et al., 2023). The annual costs associated with treating osteoarthritis total approximately 185.5 billion dollars (Kotlarz et al., 2009). With the growing elderly population, KOA presents significant challenges to both society’s economy and individuals' daily lives. Therefore, alleviating pain, preventing disease progression, and improving the daily functioning of patients with KOA are crucial for reducing the social and economic burden of the disease.

Weakness in knee extensor muscles is strongly associated with the worsening of pain and functional impairment in KOA (Oiestad et al., 2022; Muraki et al., 2015). To prevent a vicious cycle of escalating clinical symptoms, patients with KOA require appropriate interventions targeting both pain and muscle strength (Ruhdorfer et al., 2017). Resistance training has been shown to improve muscle strength, reduce joint loading stress, alleviate pain, and enhance overall functional capacity in these patients (Bartholdy et al., 2017; Turner et al., 2020). However, significantly improvements in osteoarthritis pain and physical function typically require 8–12 weeks of resistance training (Turner et al., 2020). For optimal muscle strength gains, the training load should reach 60%–70% of the one-repetition maximum (1RM) (American College of Sports Medicine, 2009). Excessive resistance load can increase knee joint pressure during training, making it intolerable for long-term patients with KOA and knee joint pain (Buford et al., 2015; Messier et al., 2021; Cassidy et al., 2023). While low-resistance training (LRT) may be better tolerated by patients with KOA, it does not provide substantial benefits over high-resistance training (HRT) in terms of improving pain, function, or quality of life, and may even be less effective in enhancing muscle strength (Hua et al., 2023; Regnaux et al., 2015; Schoenfeld et al., 2016). Consequently, there is a need to identify a treatment modality that can concurrently address pain and muscle strength.

LL-BFRT is a therapeutic approach that combines LRT with blood flow restriction therapy. By restricting blood flow to the limbs, LL-BFRT induces a hypoxic environment, promoting the accumulation of metabolites, stimulating type III and IV afferent fibers, and inhibiting alpha motor neurons, leading to recruitment of type II muscle fibers and ultimately enhancing muscle strength (Watson et al., 2022; Meyer, 2006; Rossi et al., 2018). Research has demonstrated that LL-BFRT is as effective as LRT in reducing pain and produces similar improvements in muscle strength as HRT (Peng et al., 2024; Li et al., 2021). Additionally, a study has indicated that 2 weeks of LL-BFRT has positive effects on symptoms, function, and lower limb muscle strength in patients with KOA, suggesting that LL-BFRT may be a more suitable treatment option for these patients (Kim et al., 2024). However, two previous systematic reviews have shown no significant differences in the improvement of pain, muscle strength, muscle size, and physical function between LL-BFRT and resistance training (Wang et al., 2022a; Grantham et al., 2021). This lack of significance may be attributed to the limited number of studies included and the substantial variation in intervention protocols across the included studies. Over the past 3 years, several randomized controlled studies related to LL-BFRT and KOA have been published (Hu et al., 2023; MAI et al., 2018; Dugis et al., 2023a; Dugis et al., 2023b; Pramana et al., 2023a; Pramana et al., 2023b; Sari et al., 2023; Shakeel et al., 2021). Therefore, we have incorporated these newly published studies to conduct a comprehensive systematic review and meta-analysis.

The primary objective of this study is to conduct a systematic review and meta-analysis to assess the effectiveness of LL-BFRT in the rehabilitation of patients with KOA. To this end, this study compared the effects of LL-BFRT with conventional resistance training on pain, muscle strength, and functional activity in individuals with KOA. We hypothesize that LL-BFRT will exert a more pronounced effect in improving pain, muscle strength, and functional activity than conventional resistance training.

## Materials and methods

### Search strategy

This systematic review and meta-analysis was conducted following the guidelines provided in the PRISMA statement (Prospero registration number: CRD42024603542). A search was conducted across six electronic databases, including PubMed, Embase, Web of Science, EBSCO, Scopus, and Cochrane trails. The literature search covered all relevant studies published from the inception of the databases up to 1 August 2024. The search terms for literature retrieval consisted of “knee osteoarthritis,” “blood flow restriction training,” and their synonyms. The specific search process was as follows: firstly, the search was conducted using the MeSH terms “Osteoarthritis, Knee,” and the keywords “Knee Osteoarthritides,” “Knee Osteoarthritis,” “Osteoarthritis of the Knee,” “Osteoarthritis of Knee,” and “KOA” linked with the operator “OR”. Secondly, the search was performed using the MeSH terms “Blood Flow Restriction Therapy” and the keywords “BFR Therapy,” “Blood Flow Restriction Training,” “Therapy, BFR,” “Blood Flow Restriction Exercise,” “BFR Therapies,” “Kaatsu,” “Vascular Occlusion Training,” and “Occlusion Training” linked with the operator “OR.” The results of the two search parts were then linked using the operator “AND”. The detailed search strategy is provided in Supplementary Material S1. In addition, the reference lists of similar studies were carefully reviewed to identify additional relevant articles. Two researchers (YP and XT) independently conducted the article search, with any discrepancies resolved by a third researcher (DB).

### Inclusion and exclusion criteria

All studies were selected based on inclusion and exclusion criteria formulated according to the PICOS framework. The inclusion criteria were as follows: 1. Patients with knee osteoarthritis; 2. The intervention group received low-intensity blood flow restriction training, while the control group received resistance training without blood flow restriction; 3. Outcome measures included pain-related assessment indicators (WOMAC: Western Ontario and McMaster Universities Osteoarthritis Index; VAS: Visual Analog Scale; NRS: Numerical Pain Rating Scale; KOOS: Knee Injury and Osteoarthritis Outcome Score), quadriceps strength-related indicators, and functional performance-related indicators (30STS: 30s sit to stand test; TUG: timed up and go test); 4. Randomized controlled trials; 5. publications in English. The exclusion criteria were as follows: 1. Duplicate publications; 2. Conference abstracts; 3. Full-text articles not accessible; 4. Protocols.

### Study selection and data extraction

Two reviewers (YZ and XC) independently reviewed the titles, abstracts, and full texts of retrieved articles, screened them according to inclusion and exclusion criteria, and extracted data into a pre-designed electronic spreadsheet. The extracted data included the following: 1. Publication year; 2. Sample size; 3. Age; 4. Training protocol (including exercise mode, Occlusion pressure, Exercise load, duration, frequency); 5. Outcome measures; 6. Adverse events. Data extraction focused on pain scores, quadriceps muscle strength, and functional mobility. In cases of incomplete original data, we contacted the corresponding author of the manuscript. If the author could not be reached, we used software such as GetData Graph Digitizer 2.25 to extrapolate data from graphs. When samples from different studies originated from the same institution, duplicate outcome measure were excluded from the meta-analysis. After data screening, a cross-checking process was conducted, and discrepancies were resolved through discussion or by consulting a third reviewer (DY).

### Methodological quality assessment and risk of bias

The Physiotherapy Evidence Database (PEDro) scale was used to assess the quality of the included literature (Cashin and McAuley, 2020). The PEDro scale consists of 11 items, with a total score of 10 points (the first item is not scored). Scores below 4 are considered poor, 4-5 are fair, 6-8 are good, and 9-10 are excellent. Additionally, the revised Cochrane Risk of Bias Tool for Randomized Trials (RoB-2, version 2) was used to assess potential bias across five domains: randomization, deviation from interventions, missing data, outcome measurement, and selective reporting. Each domain can be scored for low, moderate, or high bias risk (Jac et al., 2019). Quality assessment was independently conducted by two researchers (QL and JQ), with discrepant results being discussed and resolved; in cases of disputes, a third reviewer (FW) was consulted to achieve consensus.

### Statistical analysis

Meta-analysis was conducted using Stata 17 software. All data in this study were continuous variables, and a random-effects model was applied for data synthesis. Effect sizes were reported as standardized mean difference (SMD) or weighted mean difference (WMD), and 95% confidence intervals (CI) were calculated. Heterogeneity among the included studies was analyzed using a χ2 test (with a significance level of α = 0.1) and quantitatively assessed using *I* ^*2*^; *I* ^*2*^ ≥ 50% indicates moderate to high heterogeneity among studies (Higgins et al., 2003). Sensitivity analysis was performed using the leave-one-out method to assess the stability of the results and identify potential sources of heterogeneity. Subgroup analysis was performed based on the intervention protocol (occlusion pressure, training duration) and patient characteristics (age). Considering the possibility that some studies used individualized occlusion pressures, reviewers grouped samples based on the mean occlusion pressure converted to the same units. Publication bias was assessed using the Egger’s test.

## Results

### Literature screening results

**FIGURE 1:**
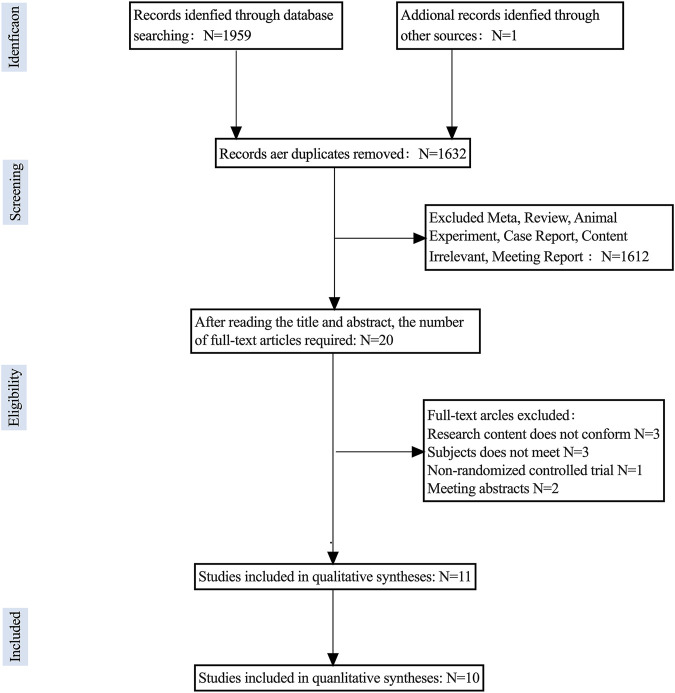
Literature Screening Flow Chart.

A total of 1959 articles were retrieved from 6 databases using relevant MeSH terms and keywords, with an additional 1 article obtained from other resources, bringing the total number of articles to 1960. After using Zotero 7 to eliminate 328 duplicate articles, the final number of articles included for review was 1,632. Upon screening titles and abstracts, a total of 1,612 articles were excluded as they were found to be systematic reviews, animal experiments, case reports, protocols, or irrelevant content, leaving 20 articles for further consideration. Following full-text review, 4 articles with incongruent research content, 1 conference abstract, 1 article with inaccessible full text, 1 non-randomized controlled trial, and 2 articles with unsuitable study subjects were excluded. Ultimately, 11 articles were selected for qualitative analysis, and 10 studies proceeded to meta-analysis. The literature screening process is depicted in Figure 1.

### Study characteristics

This study included a total of 11 studies (Hu et al., 2023; MAI et al., 2018; Dugis et al., 2023a; Dugis et al., 2023b; Pramana et al., 2023a; Pramana et al., 2023b; Sari et al., 2023; Shakeel et al., 2021; Bryk et al., 2016; Ferraz et al., 2018; Harper et al., 2019), all of which had experimental groups receiving LL-BFRT and control groups receiving conventional resistance training. 2 studies were conducted in Brazil (Bryk et al., 2016; Ferraz et al., 2018), 5 studies were conducted in Indonesia (Dugis et al., 2023a; Dugis et al., 2023b; Pramana et al., 2023a; Pramana et al., 2023b; Sari et al., 2023), 1 study was conducted in America (Harper et al., 2019), 1 study was conducted in China (Hu et al., 2023), 1 study was conducted in Egypt (MAI et al., 2018), and 1 study was conducted in Pakistan (Shakeel et al., 2021). The average age of participants in the intervention group ranged from 48.85 to 67.2 years, while the average age of participants in the control group ranged from 48.55 to 69.1 years. The intervention group consisted of 153 participants (37 males and 116 females), and the control group consisted of 174 participants (39 males and 135 females). Data from 5 studies came from the same institution (Dugis et al., 2023a; Dugis et al., 2023b; Pramana et al., 2023a; Pramana et al., 2023b; Sari et al., 2023). All studies were randomized controlled trials. Refer to Table 1 for more details.

**TABLE 1:** Study characteristics.

Study	Years	Country	Research group	Sample size (male/female)	Mean age or age range, y	Exercise mode	Exercise load	Occlusion pressure	Duration; frequency	Outcome	Adverse events
Bryk et al. (2016)	2016	Brazil	T: LL-BFRT C: HLRT	T: 17 (0/17) C: 17 (0/17)	T: 62.3 ± 7.0 C: 60.4 ± 6.7	HS; CORE training; Hip ABD and ADD; CR; CE; Sensori-motor training; KE	T:30%1RM C: 70%1RM	200 mmHg	6w, 3 days/w	NRS, Lequesne questionnaire, TUG, Quadriceps maximum isometric voluntary contraction	NR
Dugis et al. (2023a)	2023	Indonesia	T: LL-BFRT C: LIRT	T: 14 (3/11) C: 14 (2/12)	T: 57.71 ± 5.25 C: 61.42 ± 5.70	KE	T: 30%1RM C: 30%1RM	50 mmHg	6w, 2 days/w	Maximum KE torque	NR
Dugis et al. (2023b)	2023	Indonesia	T: LL-BFRT C: LIRT	T: 14 (3/11) C: 14 (2/12)	T: 57.71 ± 5.25 C: 61.42 ± 5.70	KE	T:30%1RM C: 30%1RM	50 mmHg	6w, 2 days/w	VAS	NR
Ferraz et al. (2018)	2018	Brazil	T: LL-BFRT C1: HIRT C2: LIRT	T: 16 (0/16) C1: 16 (0/16) C2: 16 (0/16)	T:59.9 ± 4 C1: 60.7 ± 4 C2: 60.3 ± 3	LP and KE	T: 30%1RM C1: 80%1RM C2: 30%1RM	70%LOP	12w, 2 days/w	1RM, 30STS, TUG, SF-36, WOMAC, Quadriceps CSA	C1:4
Harper et al. (2019)	2019	America	T: LL-BFRT C: MIRT	T: 16 (6/10) C: 19 (4/15)	T: 67.2 ± 5.2 C: 69.1 ± 7.1	LP, LE, LC, and CF	T:20%1RM C: 60%1RM	0.5(SBP)+2 (thigh circumference) + 5	12w, 3 days/w	Isokinetic strength of KE, 400 m walk gait speed, SPPB, WOMAC pain, LLFDI, Biomarkers	T: 3 C: 4
Hu et al. (2023)	2023	China	T: LL-BFRT C: RT	T: 55 (24/31) C: 57 (27/30)	T: 67.2 ± 8.2. C: 67.1 ± 7.7	SE, ROM exercise, Quadriceps muscle activation, Lower limb close chain training	NR	80%LOP	12w, NR	KOOS, ROM, 10RM, 30STS	NR
MAI et al. (2018)	2018	Egypt	T: LL-BFRT C: HLRT	T: 20 (0/20) C: 20 (0/20)	T: 48.85 ± 3.23 C: 48.55 ± 3.38	SLR,KE,Hip ABD and ADD,CR,HS	T: 30%1RM C: 60%1RM	200 mmHg	4w, 3 days/w	TUG	NR
Pramana et al. (2023a)	2023	Indonesia	T: LL-BFRT C: LIRT	T: 14 (3/11) C: 14 (2/12)	T: 57.71 ± 5.25 C: 61.42 ± 5.70	KE	T: 30%1RM C: 30%1RM	50 mmHg	6w, NR	30STS	NR
Pramana et al. (2023b)	2023	Indonesia	T: LL-BFRT C: LIRT	T:14 (3/11) C: 14 (2/12)	T: 57.71 ± 5.25 C: 61.42 ± 5.70	KE	T: 30%1RM C: 30%1RM	50 mmHg	6w, NR	JPS, TTDPM	NR
Sari et al. (2023)	2023	Indonesia	T: LL-BFRT C: LIRT	T: 14 (3/11) C: 14 (2/12)	T: 57.71 ± 5.25 C: 61.42 ± 5.70	KE	T: 30%1RM C: 30%1RM	50 mmHg	6w, NR	WOMAC	NR
Shakeel et al. (2021)	2021	Pakistan	T: LL-BFRT C: RT	T: 15 (4/11) C: 15 (6/9)	66.5 ± 6.5	Strengthening exercises of quadriceps, Hamstrings and calf muscles	NR	NR	4w, 4 days/w	VAS, Kujala scoring questionnaire, Muscle girth measurement	NR

LL-BFRT, Low-Load Blood Flow Restriction Training; LIRT, Low-Intensity Resistance Training; MIRT, Moderate-intensity resistance training; HIRT, High-intensity Resistance Training; RT, resistance training; T, treatment group; C, control group; HS, hamstring muscle stretch; KE, knee extension; SLR, straight leg raises; ABD, abduction; ADD, adduction; CORE, training: Bridge with isometric contraction of the transversus abdominis; CR, calf raises; CE, calm exercises; LP, leg press; LE, leg extension; LC, leg curl; CF, calf flexion; SE, stretching exercise; NR, no report; LOP, limb occlusion pressure; NRS, numerical pain rating scale; 30STS, 30s sit to stand test; TUG, timed up and go test; VAS, visual analog scale; RM, repetition maximum; WOMAC, Western Ontario and McMaster Universities Osteoarthritis Index; SBP, systolic blood pressure; CSA, cross-sectional area; SF-36, Short Form Health Survey; SPPB, short physical performance battery; LLFDI, the late life function and disability instrument; ROM, range of motion; KOOS, knee injury and osteoarthritis outcome score; JPS, joint position sense; TTDPM, Threshold to detect passive motion.

### Intervention protocol

All 11 studies included knee joint muscle strength training (Hu et al., 2023; MAI et al., 2018; Dugis et al., 2023a; Dugis et al., 2023b; Pramana et al., 2023a; Pramana et al., 2023b; Sari et al., 2023; Shakeel et al., 2021; Bryk et al., 2016; Ferraz et al., 2018; Harper et al., 2019). Three studies also incorporated hip joint muscle strength training and stretching exercises (Hu et al., 2023; MAI et al., 2018; Bryk et al., 2016), one study included trunk core muscle group training and movement perception trainin (Bryk et al., 2016), and one study involved range of motion (ROM) training (Hu et al., 2023). In the experimental groups, 8 studies used resistance at 30% of 1RM (MAI et al., 2018; Dugis et al., 2023a; Dugis et al., 2023b; Pramana et al., 2023a; Pramana et al., 2023b; Sari et al., 2023; Bryk et al., 2016; Ferraz et al., 2018), one study used 20% of 1RM (Harper et al., 2019), and 2 studies did not report the resistance intensity (Hu et al., 2023; Shakeel et al., 2021). Among the control groups, 6 studies used resistance at 30% of 1RM (Dugis et al., 2023a; Dugis et al., 2023b; Pramana et al., 2023a; Pramana et al., 2023b; Sari et al., 2023; Ferraz et al., 2018), 2 studies used 60% of 1RM (MAI et al., 2018; Harper et al., 2019), one study used 70% of 1RM (Bryk et al., 2016), and one study used 80% of 1RM (Ferraz et al., 2018), while 2 studies did not report the resistance intensity (Hu et al., 2023; Shakeel et al., 2021). One study calculated occlusion pressure using a formula (Harper et al., 2019), one study used 70% of limb occlusion pressure (LOP) with a mean value of 97.4 mmHg, (Ferraz et al., 2018), and one study used 80% of LOP (Hu et al., 2023). Five studies used 50 mmHg as the occlusion pressure (Dugis et al., 2023a; Dugis et al., 2023b; Pramana et al., 2023a; Pramana et al., 2023b; Sari et al., 2023), 2 studies used 200 mmHg (MAI et al., 2018; Bryk et al., 2016), and one study did not report occlusion pressure (Shakeel et al., 2021). The duration of training in 2 studies was 4 weeks (MAI et al., 2018; Shakeel et al., 2021), 6 studies trained for 6 weeks (Dugis et al., 2023a; Dugis et al., 2023b; Pramana et al., 2023a; Pramana et al., 2023b; Sari et al., 2023; Bryk et al., 2016), and 3 studies trained for 12 weeks (Hu et al., 2023; Ferraz et al., 2018; Harper et al., 2019). Training frequency varied, with 3 studies reporting training twice a week (Dugis et al., 2023a; Dugis et al., 2023b; Ferraz et al., 2018), 3 studies three times a week (MAI et al., 2018; Bryk et al., 2016; Harper et al., 2019), one study training four times a week (Shakeel et al., 2021), and 4 studies not reporting training frequency (Hu et al., 2023; Pramana et al., 2023a; Pramana et al., 2023b; Sari et al., 2023). Refer to Table 1 for more details.

### Outcome measures

Seven studies evaluated the degree of knee joint pain, with 3 studies using WOMAC pain (Sari et al., 2023; Ferraz et al., 2018; Harper et al., 2019), 2 studies using VAS, (Dugis et al., 2023b; Shakeel et al., 2021), 1 study using NRS (Bryk et al., 2016), and 1 study using KOOS pain (Hu et al., 2023). Two studies had data from the same sample (Dugis et al., 2023b; Sari et al., 2023). Four studies compared the maximum muscle strength of the quadriceps, with 2 studies assessing isokinetic peak torque (Dugis et al., 2023a; Harper et al., 2019), 1 study evaluating maximum isometric muscle strength (Bryk et al., 2016), and 1 study assessing 1RM (Ferraz et al., 2018). Three studies evaluated 30-STS (Hu et al., 2023; Pramana et al., 2023a; Ferraz et al., 2018). Three studies assessed TUG (MAI et al., 2018; Bryk et al., 2016; Ferraz et al., 2018), 1 study assessed angle reproduction difference (Pramana et al., 2023b), and 1 study assessed 400 m walking speed (Harper et al., 2019). Refer to Table 1 for more details.

### Adverse events

Two studies reported adverse events during the training process, with most of the adverse events occurring in the high-load training group (Ferraz et al., 2018; Harper et al., 2019). One study reported an exacerbation of pain related to LL-BFRT in three cases (Harper et al., 2019). Additionally, nine studies did not report any adverse events. Refer to Table 1 for more details.

### Meta-analysis results

#### Effects of LL-BFRT on pain

**FIGURE 2:**
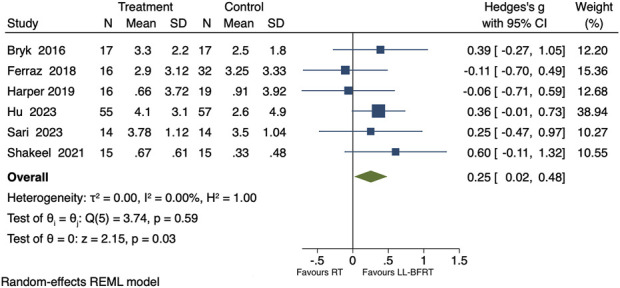
Forest plot of the impact of LL-BFRT on pain.

Included in the study were 6 comparisons of the effects of LL-BFRT and conventional resistance training on knee joint pain, with a total of 287 participants (Hu et al., 2023; Sari et al., 2023; Shakeel et al., 2021; Bryk et al., 2016; Ferraz et al., 2018; Harper et al., 2019). The overall analysis revealed low heterogeneity across the 6 studies (p = 0.59, I^2^ = 0%), indicating that LL-BFRT significantly alleviated knee joint pain [SMD = 0.25, 95%CI (0.02, 0.48), P = 0.03], as shown in Figure 2. Leave-one-out sensitivity analysis revealed that the results became non-significant when each of the 3 studies was removed individually, indicating instability in the results, as shown in Table 3 (Hu et al., 2023; Shakeel et al., 2021; Higgins et al., 2003). Egger’s test yielded a P value of 0.7140, indicating no significant publication bias among the 6 studies.

Subgroup analyses revealed that, for occlusion pressures ≤100 mmHg, results showed low heterogeneity (p = 0.73, I^2^ = 0%) and no significant difference in pain improvement between the groups [SMD = 0.00, 95% CI (−0.37, 0.38), p = 0.98]. For occlusion pressures >100 mmHg, results showed low heterogeneity (p = 0.84, I^2^ = 0%) with a significantly greater reduction in pain compared to the control group [SMD = 0.41, 95% CI (0.11, 0.70), p = 0.01]. For a training duration of ≤6 weeks, results showed low heterogeneity (p = 0.79, I^2^ = 0%) with more significant pain improvement compared to the control group [SMD = 0.41, 95% CI (0.01, 0.82), p = 0.04]. In contrast, for a training duration >6 weeks, results showed low heterogeneity (p = 0.31, I^2^ = 25.27%) and no significant pain improvement between groups [SMD = 0.14, 95% CI (−0.20, 0.48), p = 0.42]. For individuals aged ≤65 years, results showed low heterogeneity (p = 0.52, I^2^ = 0%) and no significant difference in pain improvement between the groups [SMD = 0.15, 95% CI (−0.23, 0.53), p = 0.43]. For individuals aged >65 years, results showed low heterogeneity (p = 0.37, I^2^ = 0%) with a significantly greater reduction in pain compared to the control group [SMD = 0.32, 95% CI (0.02, 0.61), p = 0.03], as shown in Table 2.

**TABLE 2:** Summary table of overall and subgroup analysis results.

Outcomes	Overall and subgroup analysis		No. of study	Sample size	MD (95% CI)	P-value	Heterogeneity	
							*I* ^***2***^	P-value
Pain	Overall		6	287	0.25 (0.02, 0.48)	0.03	0.00%	0.59
	**Subgroup**	≤100 mmHg	3	111	0.00 (−0.37, 0.38)	0.98	0.00%	0.73
		>100 mmHg	3	176	0.41 (0.11, 0.70)	0.01	0.00%	0.84
		≤6 week	3	92	0.41(0.01, 0.82)	0.04	0.00%	0.79
		>6 weeks	3	195	0.14(−0.20,0.48)	0.42	25.27%	0.31
		≤65 years	3	110	0.15 (−0.23, 0.53)	0.43	0.00%	0.52
		>65 years	3	177	0.32 (0.02. 0.61)	0.03	0.00%	0.37
**Strength**	**Overall**		4	145	0.46 (0.04, 0.88)	0.03	38.05%	0.18
	**Subgroup**	≤100 mmHg	3	111	0.34 (−0.14, 0.83)	0.16	37.17%	0.19
		>100 mmHg	1	34	0.82 (0.14, 1.50)	0.02	—	—
		≤6 weeks	2	62	0.87 (0.36, 1.38)	<0.01	0.00%	0.83
		>6 weeks	2	83	0.12 (−0.32, 0.56)	0.59	0.00%	0.8
		≤65 years	3	110	0.60 (0.11, 1.09)	0.02	37.25%	0.21
		>65 years	1	35	0.06 (−0.59, 0.71)	0.86	—	—
**30STS**	**Overall**		3	188	1.71 (0.30, 3.11)	0.02	71.79%	0.06
	**Subgroup**	≤100 mmHg	2	76	1.29 (−1.52, 4.09)	0.37	79.91%	0.03
		>100 mmHg	1	112	2.10 (1.63, 2.57)	<0.01	—	—
		≤6 weeks	1	28	2.64 (1.13, 4.15)	<0.01	—	—
		>6 weeks	2	160	1.15 (−1.09, 3.39)	0.31	79.39%	0.03
		≤65 years	2	76	0.57 (−0.73, 1.87)	0.39	85.57%	0.01
		>65 years	1	112	1.65 (1.23, 2.08)	<0.01	—	—
**TUG**	**Overall**		3	122	−0.13 (−0.51,0.24)	0.49	0.00%	0.97
	**Subgroup**	≤100 mmHg	1	48	−0.15 (−0.62,0.32)	0.53	—	—
		>100 mmHg	2	74	−0.10 (−0.73, 0.53)	0.75	0.00%	0.83
		≤6 weeks	2	74	−0.10 (−0.73,0.53)	0.75	0.00%	0.83
		>6 weeks	1	48	−0.15 (−0.62,0.32)	0.53	—	—

30STS, 30s sit to stand test; TUG, timed up and go test; MD, mean difference; CI, confidence interval.

#### Effect of LL-BFRT on quadriceps muscle strength

**FIGURE 3:**
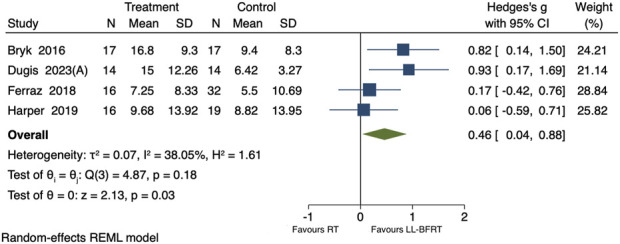
Forest plot of the impact of LL-BFRT on quadriceps muscle strength.

Including 4 studies comparing LL-BFRT and conventional resistance training on the maximal quadriceps muscle strength, with a total of 145 participants (Dugis et al., 2023a; Bryk et al., 2016; Ferraz et al., 2018; Harper et al., 2019). The overall analysis revealed low heterogeneity across the 4 studies (p = 0.18, *I* ^*2*^ = 38.05%). LL-BFRT was found to significantly improve quadriceps muscle strength [SMD = 0.46, 95%CI (0.04, 0.88), P = 0.03], as shown in Figure 3. Leave-one-out sensitivity analysis revealed that the results became non-significant when each of the 2 studies was removed individually, indicating instability in the results, as shown in Table 3 (Dugis et al., 2023a; Bryk et al., 2016). Egger’s test yielded a P value of 0.07, indicating that there was no significant publication bias among the 4 studies.

**TABLE 3:** Leave-one-out sensitivity analysis results.

Outcomes	Omitted	MD (95% CI)	P-value
Pain			
	Bryk et al. (2016)	0.23 (-0.01,0.48)	0.063
	Ferraz et al. (2018)	0.32(0.07,0.57)	0.013
	Harper et al. (2019)	0.30 (0.05,0.55)	0.018
	Hu et al. (2023)	0.18(-0.11,0.48)	0.223
	Sari et al. (2023)	0.25 (0.01,0.50)	0.042
	Shakeel et al. (2021)	0.21 (-0.03,0.46)	0.089
Strength			
	Bryk et al. (2016)	0.34 (-0.14,0.83)	0.162
	Dugis et al. (2023a)	0.33 (-0.11,0.77)	0.137
	Ferraz et al. (2018)	0.58 (0.03,1.13)	0.038
	Harper et al. (2019)	0.60 (0.11,1.09)	0.017
30STS			
	Ferraz et al. (2018)	2.15 (1.70,2.59)	<0.01
	Hu et al. (2023)	1.29 (-1.52,4.09)	0.369
	Pramana et al. (2023a)	1.15 (-1.09,3.39)	0.315

30STS, 30s sit to stand test; MD, mean difference; CI, confidence interval.

Subgroup analyses revealed that, for occlusion pressures ≤100 mmHg, results showed low heterogeneity (p = 0.19, I^2^ = 37.17%) and no significant improvement in quadriceps strength between the groups [SMD = 0.34, 95% CI (−0.14, 0.83), p = 0.16]. For occlusion pressures >100 mmHg, results showed a significantly greater improvement in quadriceps strength compared to the control group [SMD = 0.82, 95% CI (0.14, 1.50), p = 0.02]. For a training duration of ≤6 weeks, results showed low heterogeneity (p = 0.83, I^2^ = 0%) with a significant improvement in quadriceps strength compared to the control group [SMD = 0.87, 95% CI (0.36, 1.38), p < 0.01]. For studies with a training duration >6 weeks, results showed low heterogeneity (p = 0.80, I^2^ = 0%) but no significant difference in quadriceps strength between the groups [SMD = 0.12, 95% CI (−0.32, 0.56), p = 0.59]. For individuals aged ≤65 years, results showed low heterogeneity (p = 0.21, I^2^ = 37.25%) with significantly greater improvement in quadriceps strength compared to the control group [SMD = 0.60, 95% CI (0.11, 1.09), p = 0.02]. For individuals aged >65 years, results showed no significant difference in quadriceps strength improvement between the groups [SMD = 0.06, 95% CI (−0.59, 0.71), p = 0.86]. , as shown in Table 2.

#### Effect of LL-BFRT on 30STS

**FIGURE 4:**
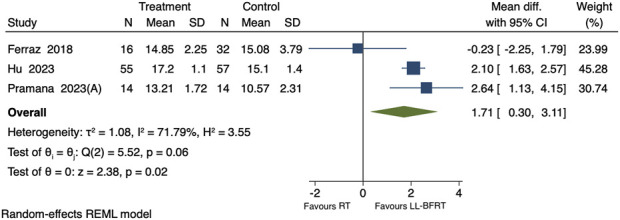
Forest plot of the impact of LL-BFRT on 30STS.

Including 3 studies comparing the effects of LL-BFRT and conventional resistance training on 30STS, involving a total of 188 participants (Hu et al., 2023; Pramana et al., 2023a; Ferraz et al., 2018). The overall analysis revealed high heterogeneity among the 3 studies (p = 0.06, *I* ^*2*^ = 71.79%). LL-BFRT was found to significantly increase the number of 30STS repetitions compared to conventional resistance training [WMD = 1.71, 95% CI (0.30, 3.11), P = 0.02], as shown in Figure 4. Leave-one-out sensitivity analysis revealed that the results became non-significant when each of the 2 studies was removed individually, indicating instability in the results, as shown in Table 3 (Hu et al., 2023; Pramana et al., 2023a). Egger’s test yielded a P value of 0.4579, indicating that there was no significant publication bias among the 3 studies.

Subgroup analyses revealed that, for occlusion pressures ≤100 mmHg, results showed high heterogeneity (p = 0.03, I^2^ = 79.91%) with no significant difference in 30STS performance between the groups [SMD = 1.29, 95% CI (−1.52, 4.09), p = 0.37]. For occlusion pressures >100 mmHg, results showed a significantly greater effect on 30STS performance compared to the control group [SMD = 2.10, 95% CI (0.63, 2.57), p < 0.01]. For a training duration of ≤6 weeks, results showed a significantly greater effect on 30STS performance compared to the control group [SMD = 2.64, 95% CI (1.13, 4.15), p < 0.01]. For studies with a training duration >6 weeks, results showed high heterogeneity (p = 0.03, I^2^ = 79.39%) with no significant difference in 30STS performance between the groups [SMD = 1.15, 95% CI (−1.09, 3.39), p = 0.31]. For individuals aged ≤65 years, results showed high heterogeneity (p = 0.01, I^2^ = 85.57%) with no significant difference in 30STS performance between the groups [SMD = 0.57, 95% CI (−0.73, 1.87), p = 0.39]. For individuals aged >65 years, results showed a significantly greater effect on 30STS performance compared to the control group [SMD = 1.65, 95% CI (1.23, 2.08), p < 0.01], as shown in Table 2.

#### Effect of LL-BFRT on TUG

**FIGURE 5:**
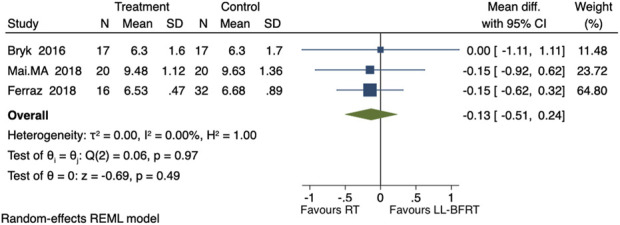
Forest plot of the impact of LL-BFRT on TUG.

Including 3 studies comparing the effects of LL-BFRT and conventional resistance training on TUG, involving a total of 122 participants (MAI et al., 2018; Bryk et al., 2016; Ferraz et al., 2018). The overall analysis revealed low heterogeneity among the 3 studies (p = 0.97, *I* ^*2*^ = 0%), indicating that LL-BFRT did not significantly reduce TUG time compared to conventional resistance training [WMD = −0.13, 95%CI (−0.51, 0.24), P = 0.49], as shown in Figure 5. Egger’s test yielded a P value of 0.8390, indicating that there was no significant publication bias among the 3 studies.

Subgroup analysis revealed that the pooled results of one study with occlusion pressure ≤100 mmHg and training duration ≤6 weeks showed no significant reduction in TUG time [SMD = −0.15, 95%CI (−0.62, 0.32), P = 0.53]. The results of two studies with occlusion pressure >100 mmHg and training duration >6 weeks demonstrated low heterogeneity between the 2 studies (p = 0.83, *I* ^*2*^ = 0%), and did not significantly shorten TUG time [SMD = −0.10, 95%CI (−0.73, 0.53), P = 0.75], as shown in Table 2.

### Methodological quality assessment and risk of bias

Among the 11 studies included, the overall PEDro scores ranged from 5 to 8, with 2 studies scoring 5 (Pramana et al., 2023a; Ferraz et al., 2018), 4 studies scoring 6 (Dugis et al., 2023b; Pramana et al., 2023b; Sari et al., 2023; Shakeel et al., 2021), 4 studies scoring 7 (Hu et al., 2023; MAI et al., 2018; Dugis et al., 2023a; Harper et al., 2019), and 1 studies scoring 8 (Bryk et al., 2016). 9 studies were rated as good quality literature (Hu et al., 2023; MAI et al., 2018; Dugis et al., 2023a; Dugis et al., 2023b; Pramana et al., 2023b; Sari et al., 2023; Shakeel et al., 2021; Bryk et al., 2016; Harper et al., 2019), while 2 studies were rated as fair quality literature (Pramana et al., 2023a; Ferraz et al., 2018). Please refer to Supplementary Material S2. The ROB2 assessment indicated that 7 studies were rated with some concerns regarding the risk of bias (Hu et al., 2023; MAI et al., 2018; Dugis et al., 2023a; Pramana et al., 2023b; Sari et al., 2023; Bryk et al., 2016; Harper et al., 2019), while 4 studies were classified as having a high risk of bias (Dugis et al., 2023a; Pramana et al., 2023a; Shakeel et al., 2021; Ferraz et al., 2018). For randomization bias, 4 studies were rated as low risk (Hu et al., 2023; MAI et al., 2018; Bryk et al., 2016; Harper et al., 2019), 6 as having some concerns (Dugis et al., 2023a; Dugis et al., 2023b; Pramana et al., 2023b; Sari et al., 2023; Shakeel et al., 2021; Ferraz et al., 2018), and 1 as high risk (Pramana et al., 2023a). Regarding bias due to deviations from intended interventions, 9 studies were rated as having some concerns (Hu et al., 2023; MAI et al., 2018; Dugis et al., 2023a; Pramana et al., 2023a; Pramana et al., 2023b; Sari et al., 2023; Bryk et al., 2016; Ferraz et al., 2018; Harper et al., 2019), and 2 as high risk (Dugis et al., 2023b; Shakeel et al., 2021). In terms of bias related to missing data, all studies were assessed as having low risk (Hu et al., 2023; MAI et al., 2018; Dugis et al., 2023a; Dugis et al., 2023b; Pramana et al., 2023a; Pramana et al., 2023b; Sari et al., 2023; Shakeel et al., 2021; Bryk et al., 2016; Ferraz et al., 2018; Harper et al., 2019). With respect to outcome measurement bias, 6 studies were rated as low risk (Hu et al., 2023; Dugis et al., 2023a; Pramana et al., 2023b; Sari et al., 2023; Shakeel et al., 2021; Bryk et al., 2016), 3 as having some concerns (MAI et al., 2018; Pramana et al., 2023a; Harper et al., 2019), and 2 as high risk (Dugis et al., 2023b; Ferraz et al., 2018). Finally, in the assessment of selective reporting bias, 10 studies were rated as low risk (Hu et al., 2023; MAI et al., 2018; Dugis et al., 2023b; Pramana et al., 2023a; Pramana et al., 2023b; Sari et al., 2023; Shakeel et al., 2021; Bryk et al., 2016; Ferraz et al., 2018; Harper et al., 2019), and 1 as having some concerns (Dugis et al., 2023a). Please refer to Supplementary Material S3.

## Discussion

The primary aim of this review and meta-analysis was to evaluate the effect of LL-BFRT compared to conventional resistance training on pain, muscle strength, and functional mobility in patients with KOA. The overall results indicate that LL-BFRT significantly improves pain, muscle strength of the quadriceps, and 30STS performance compared to conventional resistance training. Subgroup analyses of the intervention protocol indicated that, compared to traditional training, LL-BFRT with occlusion pressure >100 mmHg and a training duration ≤6 weeks led to significantly greater improvements in pain, muscle strength, and 30STS performance. Subgroup analysis by patient age revealed that, compared to traditional training, LL-BFRT resulted in significantly greater improvements in pain and 30STS performance for patients aged >65 years with KOA, and more significant improvement in muscle strength for patients aged ≤65 years with KOA. However, no significant differences were found in the effects of LL-BFRT and conventional resistance training on the TUG test, both in the overall and subgroup analyses. It is noteworthy that the leave-one-out sensitivity analysis revealed instability in the overall results, which may arise from heterogeneity in intervention protocols, patient characteristics, and study quality among the 10 included studies. Therefore, the results of this study should be interpreted with caution.

Previous systematic reviews have indicated that the effects of LL-BFRT on improving pain, muscle strength, and functional mobility in patients with KOA are similar to those of conventional resistance training (Li et al., 2021; Wang et al., 2022a; Grantham et al., 2021). This finding is inconsistent with the results of our study, which may be attributed to the limited number of studies included in previous systematic reviews and the inclusion of asymptomatic patients with KOA, potentially leading to an underestimation of the clinical efficacy of LL-BFRT in patients with symptomatic KOA. Furthermore, our study demonstrated that LL-BFRT exhibited superior efficacy in improving the 30STS performance compared to conventional resistance training among patients with KOA, while its effects on the TUG test were comparable between the two interventions. This observation may be attributed to the stronger correlation between the 30STS performance and both pain perception and muscle strength, suggesting that LL-BFRT not only demonstrates more pronounced efficacy in pain alleviation and muscular strengthening but also leads to significantly greater improvements in 30STS performance compared to conventional interventions (Khuna et al., 2024). The effects of LL-BFRT and conventional resistance training on improving TUG are similar, likely due to the stronger association of TUG with postural and balance functions (Türk et al., 2024). Two studies demonstrated that LL-BFRT exhibited comparable effects to conventional resistance training in improving proprioception and 400-meter walking speed among patients with KOA, indicating that LL-BFRT does not confer superior efficacy in enhancing balance function (Pramana et al., 2023b; Harper et al., 2019).

LL-BFRT may be more effective than conventional resistance training in significantly improving pain for three possible reasons. Firstly, LL-BFRT induces a conditional pain modulation effect by creating an ischemic environment through blood flow restriction, which subsequently inhibits joint pain (Fujii et al., 2006; Tuveson et al., 2006). Secondly, the exercise status during LL-BFRT and the local ischemic environment may enhance the release of substances such as nitric oxide, which contribute to inducing analgesia (Faiss et al., 2013; Galdino et al., 2015). Thirdly, blood flow restriction resistance training leads to higher levels of fatigue compared to conventional resistance training (de Queiros et al., 2023). Achieving a state of volitional fatigue post exercise may result in a decreased perception of pain (Yang et al., 2024). Higher occlusion pressure better activates muscles, induces higher levels of neuromuscular fatigue, stimulates endogenous opioid production, and enhances conditional pain modulation, resulting in a stronger and longer-lasting analgesic effect (Fatela et al., 2016; Hughes and Patterson, 2020). For patients with KOA, LL-BFRT with occlusion pressure >100 mmHg was found to significantly alleviate pain more effectively than conventional resistance training. It is important to note that age may be a limiting factor in pain improvement. Our analysis indicated that LL-BFRT was more effective in reducing pain in KOA patients aged >65 years compared to those aged ≤65 years. Considering the correlation between age and the severity of KOA, LL-BFRT may provide greater benefits for patients with more severe symptoms of KOA (Kim et al., 2016).

LL-BFRT also exhibited a more significant effect on muscle strength. This may be due to the ischemic environment and metabolite accumulation environment generated by blood flow restriction training, which enhances protein synthesis and type II muscle fiber recruitment (Vopat et al., 2020). Additionally, moderate occlusion pressure can increase cortical activity, recruit larger motor units, and elevate the neural discharge rate to enhance muscle strength output (Jia et al., 2024). A previous cross-sectional study indicated that LL-BFRT at an occlusion pressure of 70% of LOP (>100 mmHg) effectively increases quadriceps muscle strength and alleviates knee joint pain (Mahmoud et al., 2021). This finding is consistent with the conclusions of our subgroup analysis, which showed that LL-BFRT with occlusion pressure >100 mmHg was more effective than conventional resistance training in improving quadriceps strength in patients with KOA. LL-BFRT demonstrated superior effectiveness in enhancing quadriceps strength in KOA patients aged ≤65 years compared to those aged >65 years. This result aligns with the findings of a previous systematic review, which further indicated that LL-BFRT targeting lower limb muscle strength had particularly significant effects in the 55–64 age group (Li et al., 2023).

Based on a subgroup analysis by training duration, we found that when the training period was 4–6 weeks, LL-BFRT was more effective in alleviating pain and enhancing muscle strength than conventional resistance training. Previous studies have demonstrated that LL-BFRT can reduce the time required for patients to regain mobility, whereas traditional resistance training typically requires 8–12 weeks to achieve significant improvements in osteoarthritis-related pain and physical function (Turner et al., 2020; Jack et al., 2023). These findings suggest that LL-BFRT has the potential to shorten the rehabilitation period for patients with KOA. It is important to note that the clinical benefits of LL-BFRT are not limited to the treatment of KOA. This intervention may also have potential value in other conditions that require low-load training, such as early postoperative rehabilitation and osteoporosis (Hughes et al., 2017; Wang et al., 2023).

Among the 11 studies included in this systematic review, only one study reported three adverse events associated with LL-BFRT, while two studies reported adverse events related to conventional resistance training (Ferraz et al., 2018; Harper et al., 2019). These findings suggest that LL-BFRT is not associated with a higher risk of adverse events compared to conventional resistance training (Hughes et al., 2017). It is important to note that for patients with hypertension, BFRT may temporarily elevate blood pressure, potentially increasing the risk of cerebrovascular events (Zota et al., 2023). Therefore, hypertension risk screening should be conducted before using LL-BFRT. Furthermore, previous case reports have documented the occurrence of rhabdomyolysis following LL-BFRT (Clark and Manini, 2017). It is recommended that initial application of BFRT should commence with low intensity and short duration, gradually increasing to allow for adaptation. Particular attention should be paid to precursors of rhabdomyolysis, such as delayed onset muscle soreness and abnormal urine color.

This study has several limitations, and the results should be interpreted with caution. First, the control group intervention in this study was non-blood flow restricted resistance training, and comparisons between LL-BFRT and resistance training with different intensities may yield different results, potentially affecting the meta-analysis outcomes. Second, most of the included studies had small sample sizes (<40), and there were significant differences in the intervention protocols across studies, which could lead to instability in the results observed in the sensitivity analysis. Third, some studies did not provide detailed methodological descriptions of their interventions, including occlusion pressure, resistance intensity, and training frequency, which could introduce potential observer bias. Finally, all included studies were published in English, which may introduce language bias.

Future research should further optimize the intervention protocols of LL-BFRT for patients with knee osteoarthritis, particularly in terms of individualized occlusion pressure settings, resistance intensity, and training frequency. Several studies are currently exploring the effects of intermittent BFR and low-load training at different occlusion pressure levels on KOA (Cerqueira and de Brito Vieira, 2019; Hong et al., 2024; Jardim et al., 2022; Wang et al., 2022b), but the long-term effects and optimal parameters still require validation through high-quality randomized controlled trials. Future studies should focus on developing standardized LL-BFRT protocols, individualized treatment plans, and long-term follow-up to assess the sustainability of LL-BFRT in improving muscle strength and function, ultimately providing more effective rehabilitation options for patients with KOA.

## Conclusion

This systematic review and meta-analysis provide new insights into the effects of LL-BFRT on pain and muscle strength in patients with KOA. The results suggest limited evidence supporting the superior effects of LL-BFRT over conventional resistance training in reducing pain and improving muscle strength in patients with KOA. Subgroup analysis suggests that LL-BFRT may accelerate improvements in pain and muscle strength, with more significant effects when the occlusion pressure exceeds 100 mmHg.

## Data Availability

The original contributions presented in the study are included in the article/Supplementary Material, further inquiries can be directed to the corresponding author.
