# Corrigendum: Impact of low-load blood flow restriction training on knee osteoarthritis pain and muscle strength: a systematic review and meta-analysis of randomized controlled trials

**DOI:** 10.3389/fphys.2025.1609566

**Published:** 2025-04-23

**Authors:** Qiuxiang Lin, Debiao Yu, Yuping Zhang, Xiaoting Chen, Jiawei Qin, Fuchun Wu

**Affiliations:** ^1^ Department of Rehabilitation Medicine, Quanzhou First Hospital Affiliated to Fujian Medical University, Quanzhou, China; ^2^ College of Rehabilitation Medicine, Fujian University of Traditional Chinese Medicine, Fuzhou, China; ^3^ Provincial Clinical Medicine College of Fujian Medical University, Fuzhou, China; ^4^ Department of Rehabilitation Medicine, Fujian Provincial Hospital, Fuzhou, China; ^5^ Department of Rehabilitation Medicine, Fuzhou University Affiliated Provincial Hospital, Fuzhou, China; ^6^ Department of Orthopedics, Quanzhou First Hospital Affiliated to Fujian Medical University, Quanzhou, China

**Keywords:** blood flow restriction training, pain, rehabilitation, knee osteoarthritis, physical function

In the published article, there was an error in the **Funding** statement. The **Funding** statement was incorrectly written as “This study was supported by the Natural Science Foundation of Fujian Province (2021J01391), the Youth Talent Training Project of Fujian Provincial Health Commission (2020GGA001) and Joint Funds for the Innovation of Science and Technology, Fujian Province (Grant number: 2024Y9025).” The correct Funding statement appears below.

