# Supplementary material for: Impact of low-load blood flow restriction training on knee osteoarthritis pain and muscle strength: a systematic review and meta-analysis of randomized controlled trials
Source: Front Physiol. 2025 Mar 17;16:1524480. doi: 10.3389/fphys.2025.1524480 (PMC11955650; doi:10.3389/fphys.2025.1524480)

**Supplementary Material 1.** search strategy

**Pubmed (52)**

| #1 | "Osteoarthritis, Knee"[Mesh] |
| --- | --- |
| #2 | "Knee Osteoarthritides"[ALL Fields] |
| #3 | "Knee Osteoarthritis"[ALL Fields] |
| #4 | "Osteoarthritis of the Knee"[ALL Fields] |
| #5 | "Osteoarthritis of Knee"[ALL Fields] |
| #6 | "KOA"[ALL Fields] |
| #7 | #1 OR #2 OR #3 OR #4 OR #5 OR #6 |
| #8 | "Blood Flow Restriction Therapy"[Mesh] |
| #9 | "BFR Therapy"[ALL Fields] |
| #10 | "Blood Flow Restriction Training"[ALL Fields] |
| #11 | "Therapy, BFR"[ALL Fields] |
| #12 | "Blood Flow Restriction Exercise"[ALL Fields] |
| #13 | "BFR Therapies"[ALL Fields] |
| #14 | "Kaatsu"[ALL Fields] |
| #15 | "Vascular Occlusion Training"[ALL Fields] |
| #16 | "Occlusion Training"[ALL Fields] |
| #17 | #8 OR #9 OR #10 OR #11 OR #12 OR #13 OR #14 OR #15 OR #16 |
| #18 | #7 AND #17 |

**Cochrane trails(77)**

| #1 | "Osteoarthritis, Knee"[Mesh] |
| --- | --- |
| #2 | "Knee Osteoarthritides"[ALL Text] |
| #3 | "Knee Osteoarthritis"[ALL Text] |
| #4 | "Osteoarthritis of the Knee"[ALL Text] |
| #5 | "Osteoarthritis of Knee"[ALL Text] |
| #6 | "KOA"[ALL Text] |
| #7 | #1 OR #2 OR #3 OR #4 OR #5 OR #6 |
| #8 | "Blood Flow Restriction Therapy"[Mesh] |
| #9 | "BFR Therapy"[ALL Text] |
| #10 | "Blood Flow Restriction Training"[ALL Text] |
| #11 | "Therapy, BFR"[ALL Text] |
| #12 | "Blood Flow Restriction Exercise"[ALL Text] |
| #13 | "BFR Therapies"[ALL Text] |
| #14 | "Kaatsu"[ALL Text] |
| #15 | "Vascular Occlusion Training"[ALL Text] |
| #16 | "Occlusion Training"[ALL Text] |
| #17 | #8 OR #9 OR #10 OR #11 OR #12 OR #13 OR #14 OR #15 OR #16 |
| #18 | #7 AND #17 |

**SCOPUS (1573)**

| #1 | "Osteoarthritis, Knee"[ALL Fields] |
| --- | --- |
| #2 | "Knee Osteoarthritides"[ALL Fields] |
| #3 | "Knee Osteoarthritis"[ALL Fields] |
| #4 | "Osteoarthritis of the Knee"[ALL Fields] |
| #5 | "Osteoarthritis of Knee"[ALL Fields] |
| #6 | "KOA"[ALL Fields] |
| #7 | #1 OR #2 OR #3 OR #4 OR #5 OR #6 |
| #8 | "Blood Flow Restriction Therapy"[ALL Fields] |
| #9 | "BFR Therapy"[ALL Fields] |
| #10 | "Blood Flow Restriction Training"[ALL Fields] |
| #11 | "Therapy, BFR"[ALL Fields] |
| #12 | "Blood Flow Restriction Exercise"[ALL Fields] |
| #13 | "BFR Therapies"[ALL Fields] |
| #14 | "Kaatsu"[ALL Fields] |
| #15 | "Vascular Occlusion Training"[ALL Fields] |
| #16 | "Occlusion Training"[ALL Fields] |
| #17 | #8 OR #9 OR #10 OR #11 OR #12 OR #13 OR #14 OR #15 OR #16 |
| #18 | #7 AND #17 |

**EBSCO (MEDLINE) (88)**

| #1 | "Osteoarthritis, Knee"[TX] |
| --- | --- |
| #2 | "Knee Osteoarthritides"[TX] |
| #3 | "Knee Osteoarthritis"[TX] |
| #4 | "Osteoarthritis of the Knee"[TX] |
| #5 | "Osteoarthritis of Knee"[TX] |
| #6 | "KOA"[TX] |
| #7 | #1 OR #2 OR #3 OR #4 OR #5 OR #6 |
| #8 | "Blood Flow Restriction Therapy"[TX] |
| #9 | "BFR Therapy"[TX] |
| #10 | "Blood Flow Restriction Training"[TX] |
| #11 | "Therapy, BFR"[TX] |
| #12 | "Blood Flow Restriction Exercise"[TX] |
| #13 | "BFR Therapies"[TX] |
| #14 | "Kaatsu"[TX] |
| #15 | "Vascular Occlusion Training"[TX] |
| #16 | "Occlusion Training"[TX] |
| #17 | #8 OR #9 OR #10 OR #11 OR #12 OR #13 OR #14 OR #15 OR #16 |
| #18 | #7 AND #17 |

**Web of Science (91)**

| #1 | (((((TS=(Osteoarthritis, Knee)) OR TS=(Knee Osteoarthritides)) OR TS=(Knee Osteoarthritis)) OR TS=(Osteoarthritis of the Knee)) OR TS=(Osteoarthritis of Knee)) OR TS=(KOA) |
| --- | --- |
| #2 | ((((((TS=(Blood Flow Restriction Therapy)) OR TS=(BFR Therapy)) OR TS=(Blood Flow Restriction Training)) OR TS=(Therapy, BFR)) OR TS=(Blood Flow Restriction Exercise)) OR TS=(BFR Therapies)) OR TS=(Kaatsu) |
| #3 | #1 and #2 |

**Embase (78)**

| #1 | "Osteoarthritis, Knee"[Broad search] |
| --- | --- |
| #2 | "Knee Osteoarthritides"[Broad search] |
| #3 | "Knee Osteoarthritis"[Broad search] |
| #4 | "Osteoarthritis of the Knee"[Broad search] |
| #5 | "Osteoarthritis of Knee"[Broad search] |
| #6 | "KOA"[Broad search] |
| #7 | #1 OR #2 OR #3 OR #4 OR #5 OR #6 |
| #8 | "Blood Flow Restriction Therapy"[Broad search] |
| #9 | "BFR Therapy"[Broad search] |
| #10 | "Blood Flow Restriction Training"[Broad search] |
| #11 | "Therapy, BFR"[Broad search] |
| #12 | "Blood Flow Restriction Exercise"[Broad search] |
| #13 | "BFR Therapies"[Broad search] |
| #14 | "Kaatsu"[Broad search] |
| #15 | "Vascular Occlusion Training"[Broad search] |
| #16 | "Occlusion Training"[Broad search] |
| #17 | #8 OR #9 OR #10 OR #11 OR #12 OR #13 OR #14 OR #15 OR #16 |
| #18 | #7 AND #17 |

**Supplementary Material 2.**

PEDro score for Methodological Quality assessment of including studies

| Study | Item 1 | Item 2 | Item 3 | Item 4 | Item 5 | Item 6 | Item 7 | Item 8 | Item 9 | Item 10 | Item 11 | Score | Quality |
| --- | --- | --- | --- | --- | --- | --- | --- | --- | --- | --- | --- | --- | --- |
| Bryk^40^ | 1 | 1 | 1 | 1 | 0 | 0 | 1 | 1 | 1 | 1 | 1 | 8\10 | good |
| Dugis(A)^31^ | 1 | 1 | 0 | 1 | 0 | 0 | 1 | 1 | 1 | 1 | 1 | 7\10 | good |
| Dugis(B)^32^ | 1 | 1 | 0 | 1 | 0 | 0 | 0 | 1 | 1 | 1 | 1 | 6\10 | good |
| Ferraz^41^ | 1 | 1 | 0 | 0 | 0 | 0 | 0 | 1 | 1 | 1 | 1 | 5\10 | fair |
| Harper^42^ | 1 | 1 | 1 | 0 | 0 | 0 | 1 | 1 | 1 | 1 | 1 | 7\10 | good |
| Hu^29^ | 1 | 1 | 0 | 1 | 0 | 0 | 1 | 1 | 1 | 1 | 1 | 7\10 | good |
| Mai.M.A^30^ | 1 | 1 | 1 | 1 | 0 | 0 | 0 | 1 | 1 | 1 | 1 | 7\10 | good |
| Pramana(A)^33^ | 1 | 0 | 0 | 1 | 0 | 0 | 0 | 1 | 1 | 1 | 1 | 5\10 | fair |
| Pramana(B)^34^ | 1 | 1 | 0 | 1 | 0 | 0 | 0 | 1 | 1 | 1 | 1 | 6\10 | good |
| Sari^35^ | 1 | 1 | 0 | 1 | 0 | 0 | 0 | 1 | 1 | 1 | 1 | 6\10 | good |
| Shakeel^36^ | 1 | 1 | 0 | 1 | 0 | 0 | 0 | 1 | 1 | 1 | 1 | 6\10 | good |
| Item 1= eligibility criteria; Item 2 = random allocation; Item 3 = concealed allocation; Item 4 = similar baseline; Item 5 = subjected blinded; Item 6 = therapists blinded; Item 7 = assessors blinded; Item 8 = <15% dropouts; Item 9 = intention-to-treat analysis; Item 10 = between-group comparison; Item 11 = point measures and variability data; 1 = described explicitly and in details; 0 = unclear, inadequately described. | | | | | | | | | | | | | |

**Supplementary Material 3.** Rob2.Risk of bias


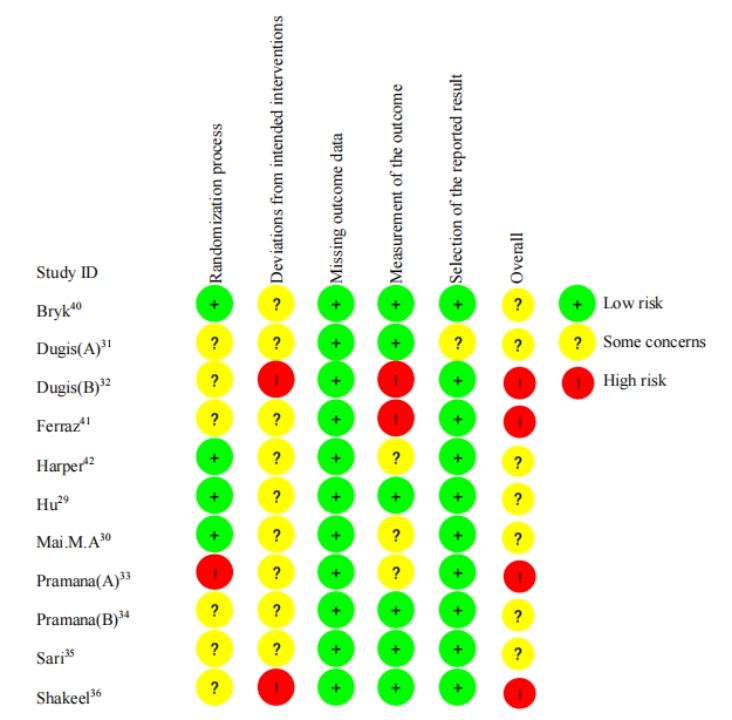

Supplement: Supplementary file 1 [file Table1.docx]
